# Supplementary material for: Child Overweight or Obesity Is Associated with Modifiable and Geographic Factors in Vietnam: Implications for Program Design and Targeting
Source: Nutrients. 2020 May 1;12(5):1286. doi: 10.3390/nu12051286 (PMC7282258; doi:10.3390/nu12051286)
Supplement: Supplementary file 1 [file nutrients-12-01286-s001.pdf]

Table S1. Bivariate relative risks of overweight or obesity among children < 5 y at the child, maternal, household, and environmental levels between 2012 and 2015

Variable	N	RR (95% CI)	P-Value
Child level			
Age, y	391852	1.07 (1.05, 1.08)	< 0.0001
Sex:			
Boy	205847	1.37 (1.32, 1.41)	< 0.0001
Girl	186005	1.00 (Reference)	
Ethnicity			
Kinh	296151	1.90 (1.78, 2.03)	< 0.0001
Minority	95701	1.00 (Reference)	
Delivery type:			
Vaginal	312506	1.00 (Reference)	
C-section	79346	1.55 (1.48, 1.61)	< 0.0001
Birthweight, g			
≤ 4000	387644	1.00 (Reference)	
> 4000	4208	2.14 (1.92, 2.40)	< 0.0001
Maternal/caregiver level			
BMI (kg/m^2)			
< 23	322742	1.00 (Reference)	
≥ 23 < 27.5	62527	1.49 (1.43, 1.55)	< 0.0001

≥ 27.5	6583	1.93 (1.75, 2.13)	< 0.0001
Education (y)			
None, primary, or secondary (grades 1–9)	228099	1.00 (Reference)	
High school (grades 10–12)	105999	1.38 (1.31, 1.44)	< 0.0001
College, university, or graduate school	57754	1.71 (1.62, 1.81)	< 0.0001
Occupation			
Farmer	163812	1.00 (Reference)	
Non-farmer	228040	2.08 (1.99, 2.18)	< 0.0001
Household level			
Number of children < 5 y in household			
≤ 2	335917	1.46 (1.38, 1.55)	< 0.0001
> 2	55935	1.00 (Reference)	
Environmental level			
Locality			
Urban	104994	1.85 (1.76, 1.94)	< 0.0001
Rural	286858	1.00 (Reference)	
Poor commune			
Yes	70512	1.00 (Reference)	
No	294138	1.55 (1.44, 1.66)	< 0.0001
Mountainous commune			

Yes	120719	1.00 (Reference)	
No	245786	1.58 (1.48, 1.68)	< 0.0001
Region			
Red River Delta	54893	1.67 (1.47, 1.89)	< 0.0001
Northeast	77689	1.80 (1.58, 2.05)	< 0.0001
Northwest	23933	1.02 (0.87, 1.21)	0.80
Central North	36633	1.41 (1.22, 1.63)	< 0.0001
Central South	35810	2.20 (1.93, 2.51)	< 0.0001
Central Highlands	24442	1.00 (Reference)	
Mekong River Delta	77720	2.12 (1.88, 2.39)	< 0.0001
Southeast	60732	3.10 (2.74, 3.51)	< 0.0001
Year	391852	1.07 (1.05, 1.10)	< 0.0001

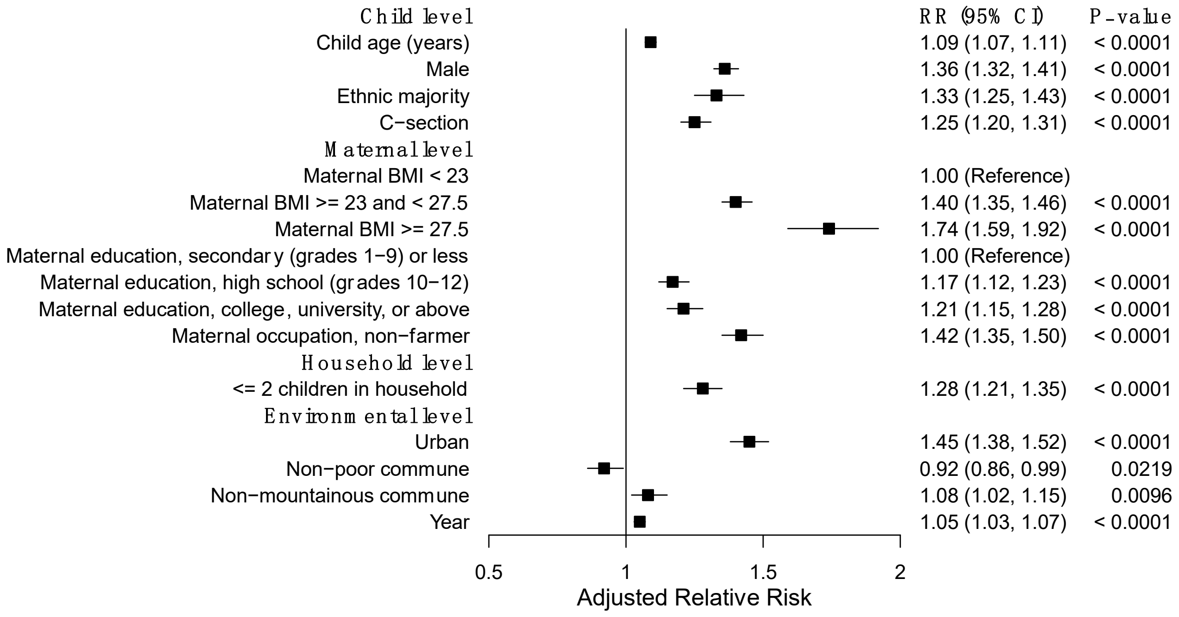

Figure S1. Relative risk of *overweight or obesity* among 391852 Vietnamese children < 5 y between 2012 and 2015. RR, relative risk; BMI, body mass index.

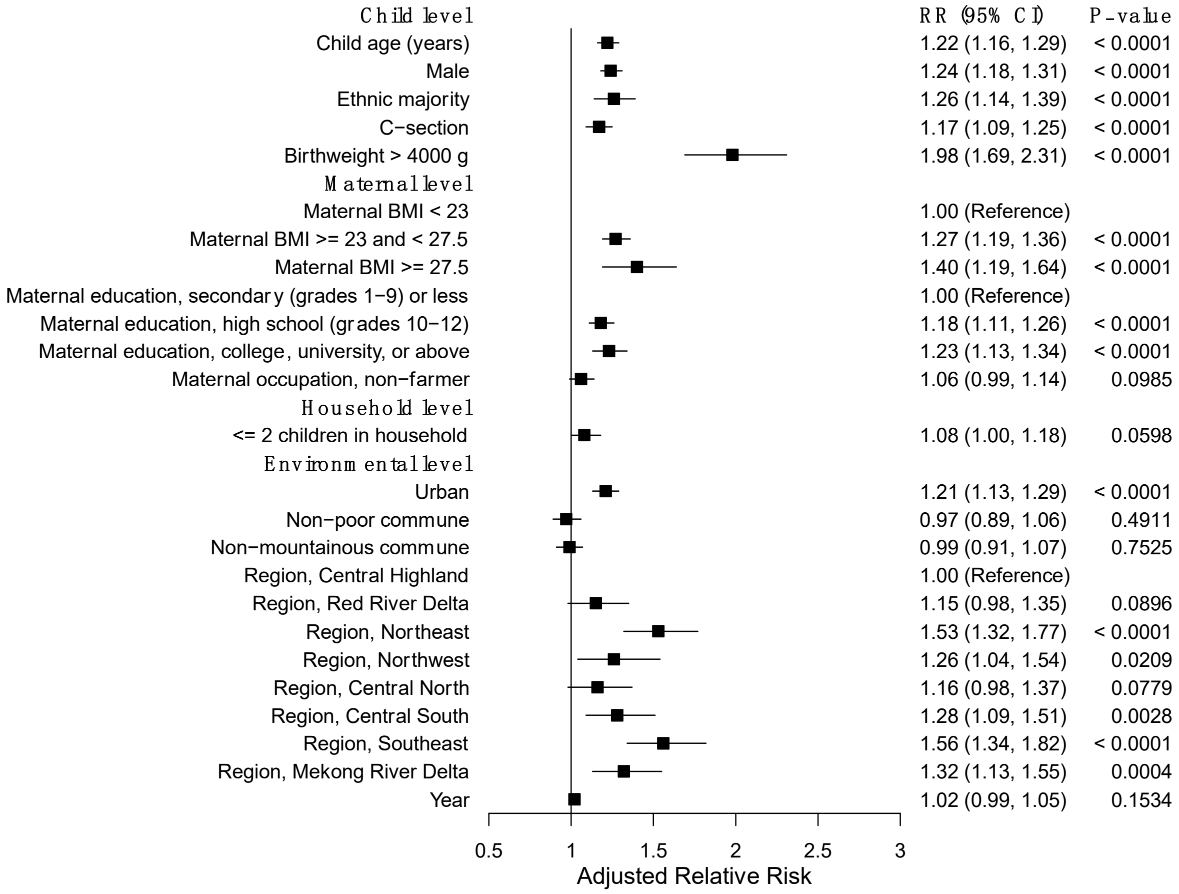

Figure S2. Relative risk of stunting among 174555 Vietnamese children 0–23 m between 2012 and 2015. RR, relative risk; g, gram; BMI, body mass index.

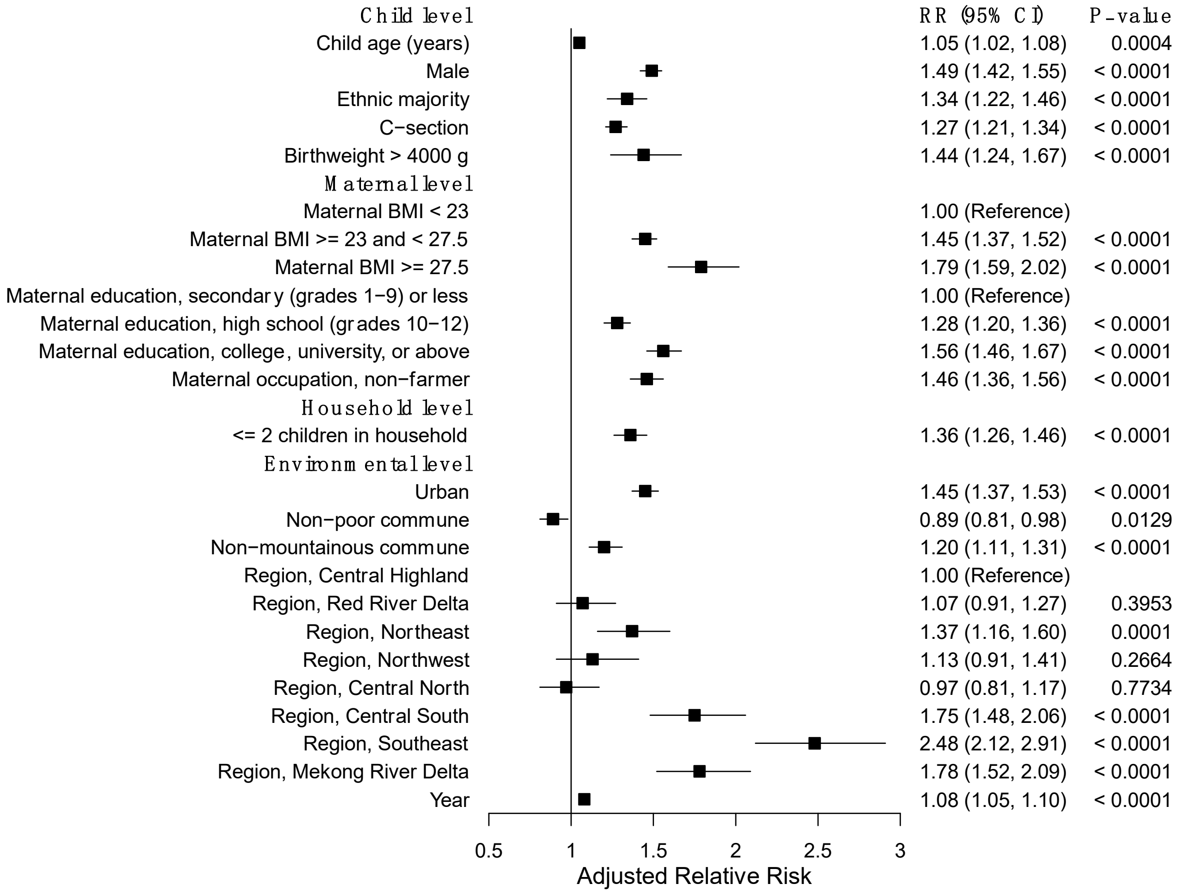

Figure S3. Relative risk of stunting among 218391 Vietnamese children 24–59 m between 2012 and 2015. RR, relative risk; g, gram; BMI, body mass index.

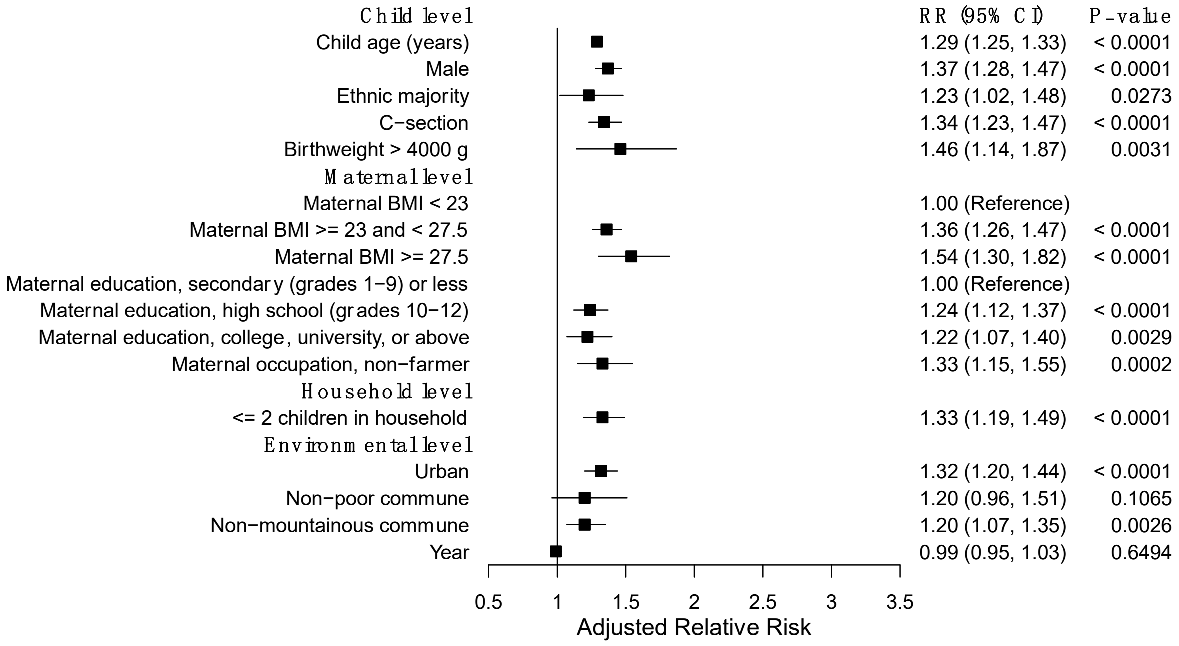

Figure S4. Relative risk of stunting among 15298 children < 5 y from the Southeast province in Vietnam between 2012 and 2015. RR, relative risk; g, gram; BMI, body mass index.

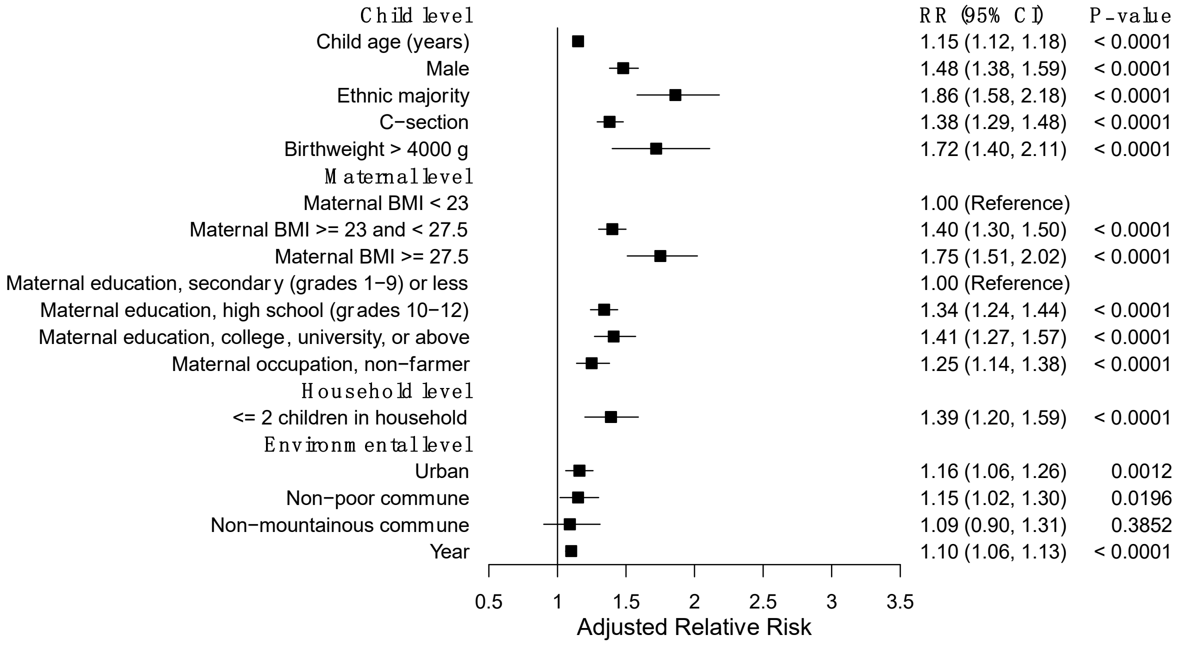

Figure S5. Relative risk of stunting among 19580 children < 5 y from the Mekong River Delta province in Vietnam between 2012 and 2015. RR, relative risk; g, gram; BMI, body mass index.

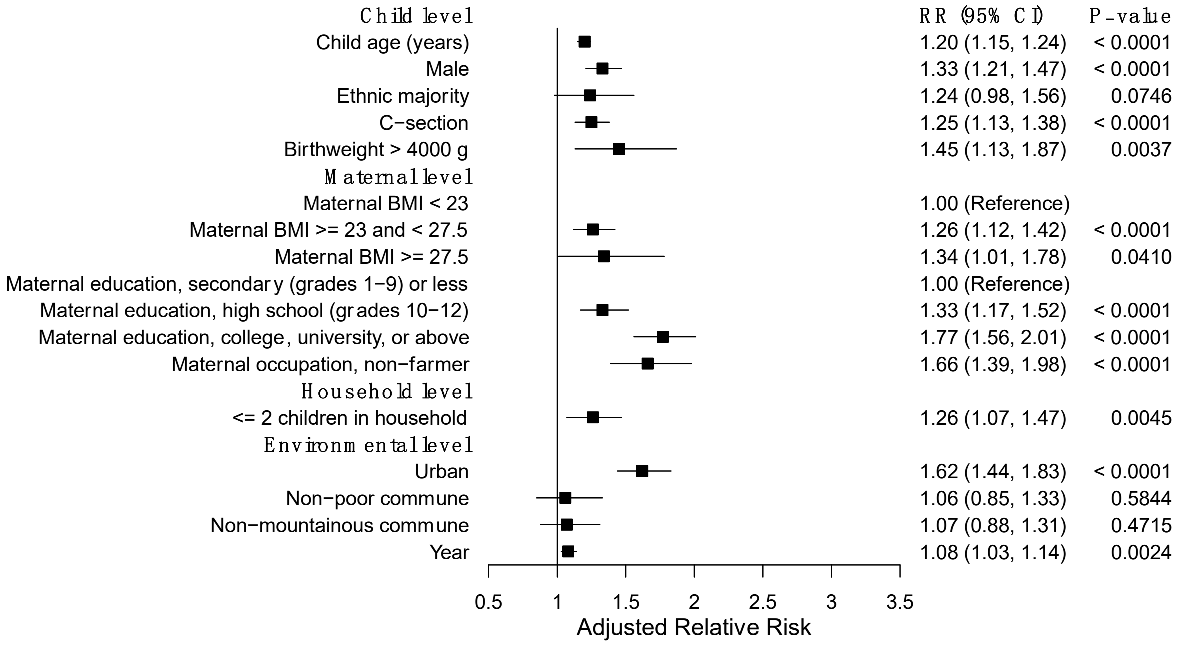

Figure S6. Relative risk of stunting among 8987 children < 5 y from the Central South province in Vietnam between 2012 and 2015. RR, relative risk; g, gram; BMI, body mass index.

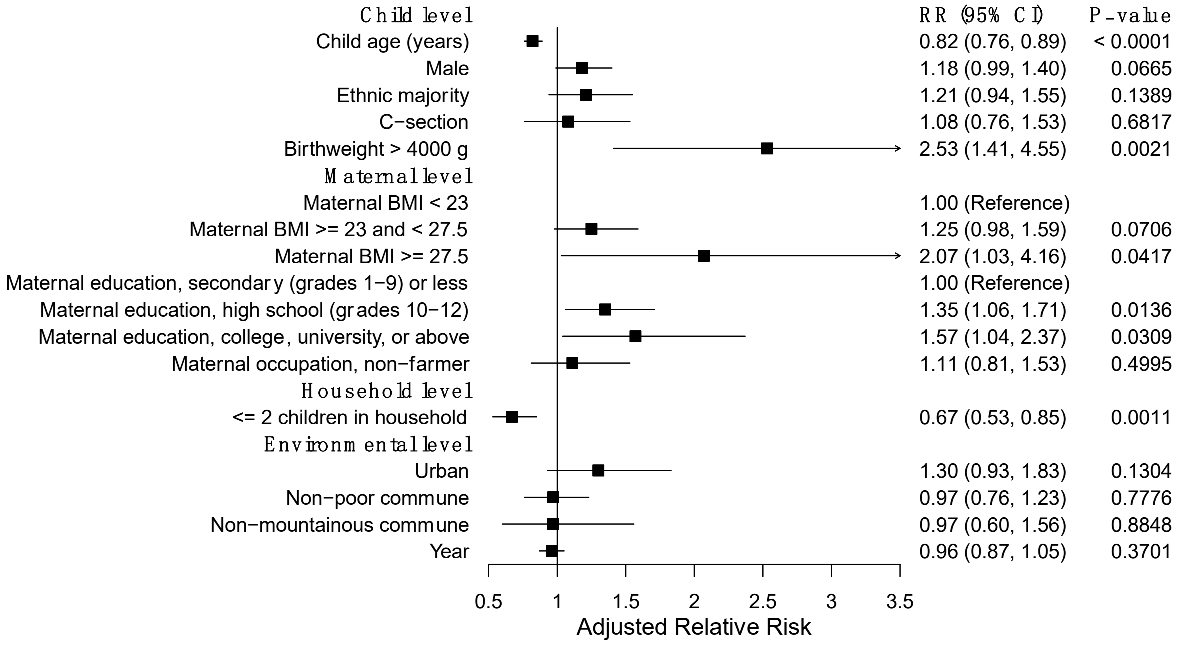

Figure S7. Relative risk of stunting among 6027 children < 5 y from the Northwest province in Vietnam between 2012 and 2015. RR, relative risk; g, gram; BMI, body mass index.

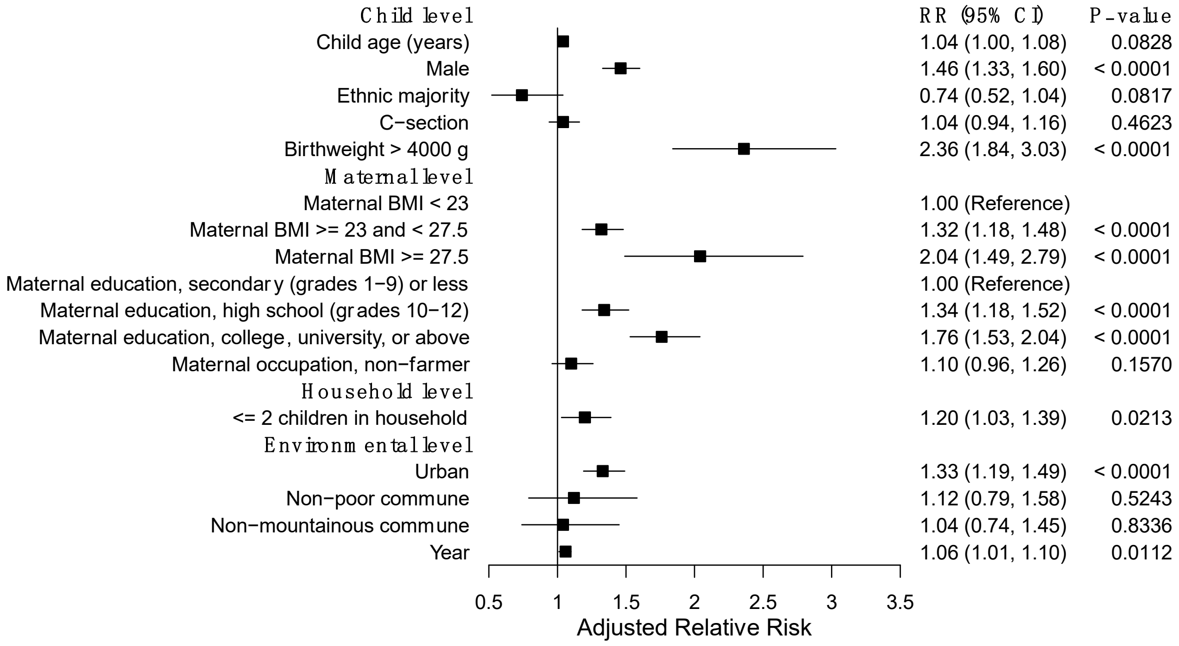

Figure S8. Relative risk of stunting among 13999 children < 5 y from the Red River Delta province in Vietnam between 2012 and 2015. RR, relative risk; g, gram; BMI, body mass index.

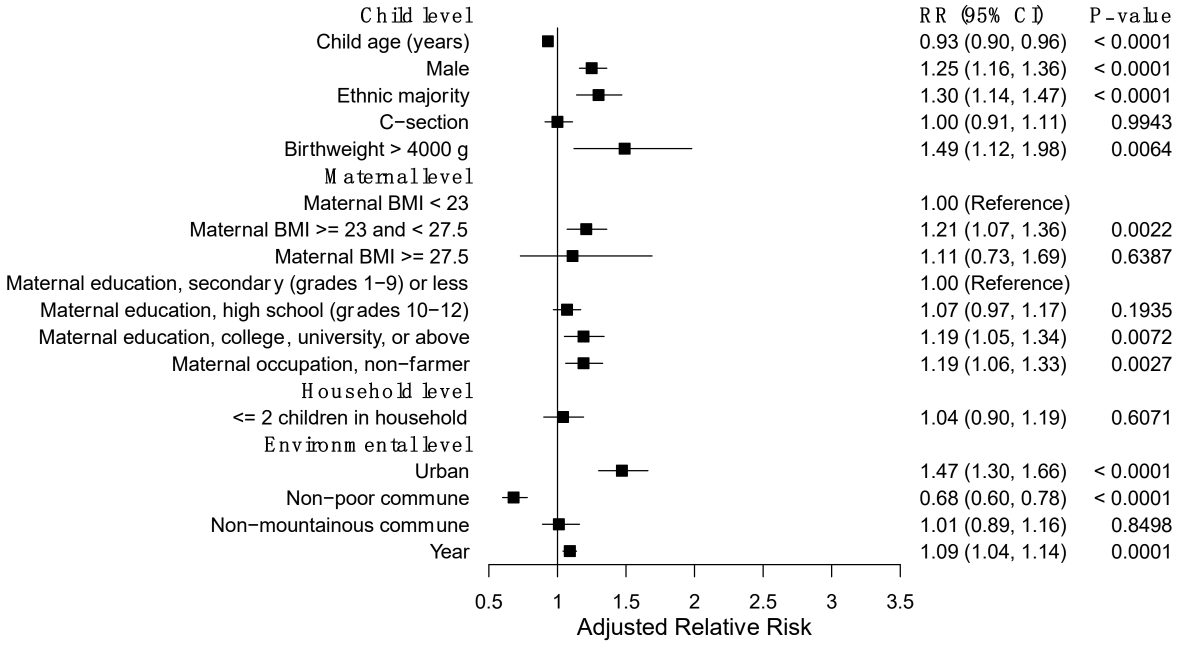

Figure S9. Relative risk of stunting among 19599 children < 5 y from the Northeast province in Vietnam between 2012 and 2015. RR, relative risk; g, gram; BMI, body mass index.
